# Recognition of Cutaneous Melanoma on Digitized Histopathological Slides *via* Artificial Intelligence Algorithm

**DOI:** 10.3389/fonc.2020.01559

**Published:** 2020-08-20

**Authors:** Francesco De Logu, Filippo Ugolini, Vincenza Maio, Sara Simi, Antonio Cossu, Daniela Massi, Italian Association for Cancer Research (AIRC) Study Group, Romina Nassini, Marco Laurino

**Affiliations:** ^1^Section of Clinical Pharmacology and Oncology, Department of Health Sciences, University of Florence, Florence, Italy; ^2^Section of Pathological Anatomy, Department of Health Sciences, University of Florence, Florence, Italy; ^3^Histopathology and Molecular Diagnostics, Careggi University Hospital, Florence, Italy; ^4^Department of Medical, Surgical, and Experimental Sciences, University of Sassari, Sassari, Italy; ^5^Institute of Clinical Physiology, National Research Council, Pisa, Italy

**Keywords:** cutaneous melanoma, artificial intelligence, convolutional neural network, image analysis, diagnosis

## Abstract

Increasing incidence of skin cancer combined with a shortage of dermatopathologists has increased the workload of pathology departments worldwide. In addition, the high intraobserver and interobserver variability in the assessment of melanocytic skin lesions can result in underestimated or overestimated diagnosis of melanoma. Thus, the development of new techniques for skin tumor diagnosis is essential to assist pathologists to standardize diagnoses and plan accurate patient treatment. Here, we describe the development of an artificial intelligence (AI) system that recognizes cutaneous melanoma from histopathological digitalized slides with clinically acceptable accuracy. Whole-slide digital images from 100 formalin-fixed paraffin-embedded primary cutaneous melanoma were used to train a convolutional neural network (CNN) based on a pretrained Inception-ResNet-v2 to accurately and automatically differentiate tumoral areas from healthy tissue. The CNN was trained by using 60 digital slides in which regions of interest (ROIs) of tumoral and healthy tissue were extracted by experienced dermatopathologists, while the other 40 slides were used as test datasets. A total of 1377 patches of healthy tissue and 2141 patches of melanoma were assessed in the training/validation set, while 791 patches of healthy tissue and 1122 patches of pathological tissue were evaluated in the test dataset. Considering the classification by expert dermatopathologists as reference, the trained deep net showed high accuracy (96.5%), sensitivity (95.7%), specificity (97.7%), F_1_ score (96.5%), and a Cohen’s kappa of 0.929. Our data show that a deep learning system can be trained to recognize melanoma samples, achieving accuracies comparable to experienced dermatopathologists. Such an approach can offer a valuable aid in improving diagnostic efficiency when expert consultation is not available, as well as reducing interobserver variability. Further studies in larger data sets are necessary to verify whether the deep learning algorithm allows subclassification of different melanoma subtypes.

## Introduction

Melanoma is one of the major causes of cancer-related death, and its incidence is increasing worldwide (1, 2). Histopathological diagnosis of melanoma is based on the assessment of cyto-architectural features on hematoxylin and eosin-stained slides, which has been recognized to be highly subjective. Recent observations have revealed a diagnostic discordance between histopathologists in distinguishing benign nevi and malignant melanomas (3, 4). A lack of access to dermatopathology expertise in this context can slow diagnostic turnaround times, resulting in delays in patient care and leading to potential adverse impacts on clinical outcomes. In this scenario, the computer-aided diagnosis (CAD) system reduces intraobserver and interobserver variability and improves the accuracy of pathology interpretation (5, 6).

In recent years, AI has attracted a lot of attention for digital imaging processing (7). The most commonly used and highly functioning AI approach for medical image processing (including histopathology) is based on deep learning algorithms and, in particular, on CNNs (8). CNNs are deep neural networks, trained for visual recognition tasks directly from pixel images with minimal preprocessing (9). CNNs require a considerable amount of data for training/validation, and the classification accuracy of CNN classifiers is mainly dependent on the quality and size of the image dataset (10). The training of a new CNN model with a new image dataset requires extensive effort to collect a large number of images. For this reason, a CNN architecture can be built from pretrained models (Transfer Learning approach) with a considerable reduction in the training image dataset (11). Several types of pretrained CNN architectures, including AlexNet, SqueezeNet, NASNet-Large, Inception-v3, ResNet-50, Vgg19, and Inception- ResNet-v2 (12–14), have been designed.

The deep learning applied to digital pathology is a continuing challenge for many reasons: (i) limited (labeled) dataset availability, (ii) complexity of pathological variability, (iii) large image sizes, and (iv) difficulty of implementation of CNN models due to a large number of setting parameters (15, 16).

In recent years, few studies that focus on deep learning algorithms have been proposed to automate the analysis of melanoma and skin lesions in WSIs (17–19). Since the sizes of WSIs are too large to be used as direct input to a CNN, the typical approach is to train, validate, and test the CNN, instead of using low-pixel-resolution patches of the WSI, obtaining tens to thousands of patches from each WSI (20). Although AI technology has achieved remarkable results for skin pathology analysis, in this field, the potential of CNNs has not been fully investigated, and their performances may be significantly improved. Future studies will focus on the development of different CNN architectures and training procedures, to finalize an optimal AI-based algorithm useful for clinical support.

In this study, we aimed to implement an annotation framework for the automated analysis of histopathological cutaneous melanoma images. We developed a CNN-based algorithm that allows us to build masks on scanned lesioned tissues, in order to define areas of healthy and pathological tissue, even in those samples for which identification by the pathologist is more complex due to the presence of a scarce tumoral component. Our data revealed a methodological approach to build a map reporting the topological distribution of melanoma and healthy tissues from analyzed WSIs.

## Materials and Methods

### Sample Population and Data Set

**FIGURE 1:**
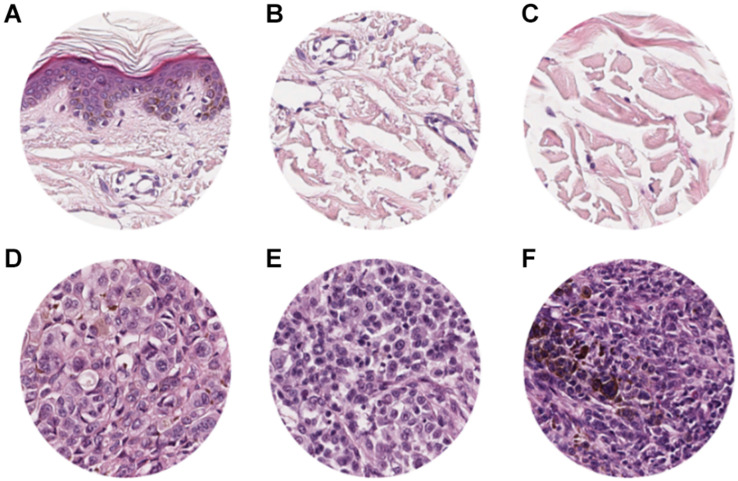
ROI samples selected from healthy tissue **(A–C)** and invasive cutaneous melanoma **(D–F)** on a whole slide image. Each ROI was obtained at a magnification of 20×.

The study included a retrospective collection of formalin-fixed paraffin-embedded (FFPE) cutaneous primary invasive melanomas (*n* = 100) from the Section of Pathology, Department of Health Sciences, University of Florence, Florence, Italy; the Center for Immuno-Oncology, Department of Oncology, University Hospital of Siena, Siena, Italy; and the Unit of Cancer Genetics, Institute of Biomolecular Chemistry (ICB), National Research Council (CNR), Sassari, Italy. Clinicopathological data of the patients are reported in Table 1. Two expert dermatopathologists (VM and DM) performed the histopathological reevaluation and confirmed the original diagnosis. Representative histopathological whole slides stained with hematoxylin and eosin were anonymized and digitalized using Pannoramic 250 Flash III (3D HISTECH) and Aperio AT2 (Leica) with × 20 power. From each scanned slide, a total of 8 ROIs (4 representatives of the tumor area and 4 of the adjacent healthy tissue) were extracted (Figure 1). The use of FFPE sections of human samples was approved by the Local Ethics Committee (#13676_bio and #17033_bio) according to the Helsinki Declaration.

**TABLE 1:** Clinicopathological data of the patients.

Patients (*n* = 100)		
**Age**	Range (Mean ± SD)	24–89 (62.7 ±16.0)
**Sex**	Male (*n*)	62
	Female (*n*)	38
**Location of primary tumor**	Trunk (*n*)	56
	Extremity (n)	44
**Tumor thickness**	>2mm (*n*)	100
**Clark’s level**	III (*n*)	47
	IV (n)	53
**Ulceration**	Present (*n*)	71
	Not present (*n*)	29
**Stage**	III (*n*)	47
	IV (*n*)	53
**Mitotic rate/mm^2^**	Range	0–57

### AI Methodology

**FIGURE 2:**
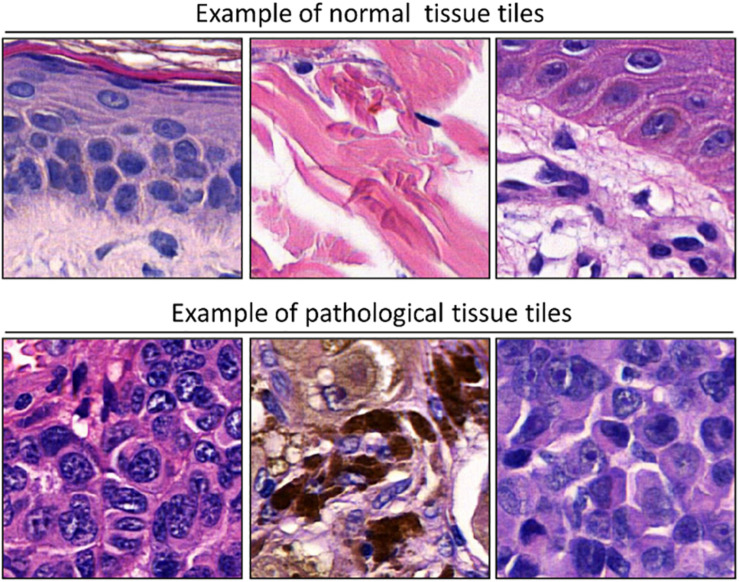
Examples of patches cropped from ROIs. The first and second lines report sampled patches of normal and pathological tissues, respectively. Each patch has a size of 299 × 299 pixels (72.59 mm × 72.59 mm).

To adapt the data size of the input images to the CNN input layer size, smaller image patches were extracted from the labeled ROI. Each ROI was tiled in non-overlapping 299 × 299 pixel square patches (×20 magnification). The patch dimension of each pixel was about 0.2428 mm; therefore, the dimension of each patch was about 72.59 mm × 72.59 mm, corresponding to an area of about 5269.3 mm^2^ (Figure 2). Patches were adjacent to each other and covered the entire tissue region of each ROI. Patches of the ROIs containing no more than 50% white background were used for further analysis. The patches obtained from ROIs of 60 slides (1377 patches for healthy tissue and 2141 patches for melanoma) were used as training/validation dataset; the patches from ROIs of 40 slides (791 for healthy tissue and 1122 patches for melanoma) were used as a test dataset. The training/validation and testing datasets were completely disjointed (i.e., extracted from different patients) in order to demonstrate the robustness of the trained CNN. The expansion of the image dataset for training and validation is useful for improving the capability of the model and to avoid overfitting. We augmented the training/validation dataset *via* affine transformations. The patches were horizontally or vertically shifted between 0 and 30 pixels and scaled by zoom-in and zoom-out operations with rate magnitude between 0 and 20%. The increased and original patches were used as training/validation sets.

A CNN based on a pretrained Inception-ResNet-v2 (12) with the training/validation dataset was trained by using 75% of the patches for training and 25% for validation. For training the net, stochastic gradient descent with momentum algorithm was used; the first 10 layers of the net were frozen, and for the residual layers, the learning rate was set to 0.003, and the momentum was set to 0.9. The maximum number of epochs for training was set to 15, and a mini-batch with 16 observations at each iteration was used. Then, the net performance with the test dataset was tested.

For binary (healthy tissue vs. melanoma) patch-level classification, the trained net performance was evaluated in terms of the following metrics:

1. Accuracy: the ratio between the number of correct predictions (both true positives and true negatives) and the overall number of samples.
2. Sensitivity (or recall): the number of true positives divided by the number of true positives and false negatives.
3. Specificity: the number of true negatives divided by the number of true negatives and false positives.
4. F_1_ score: a measure of a classification accuracy often used in case of imbalanced data. This is the harmonic mean between precision and sensitivity. Precision is the number of true positives divided by the number of true positives and false positives.
5. Cohen’s kappa: a measure of the agreement between the human and trained net classification (correcting for chance agreement). This is a statistical measure of inter-rater agreement. Kappa scores less than zero are interpreted as “no” agreement. Kappa scores ranging from 0.01 to 0.20 are considered “slight” agreement, 0.21 to 0.40 “fair” agreement, 0.41 to 0.60 “moderate” agreement, 0.61 to 0.80 “substantial” agreement, and 0.81 to 1.00 “almost perfect” agreement.

**FIGURE 3:**
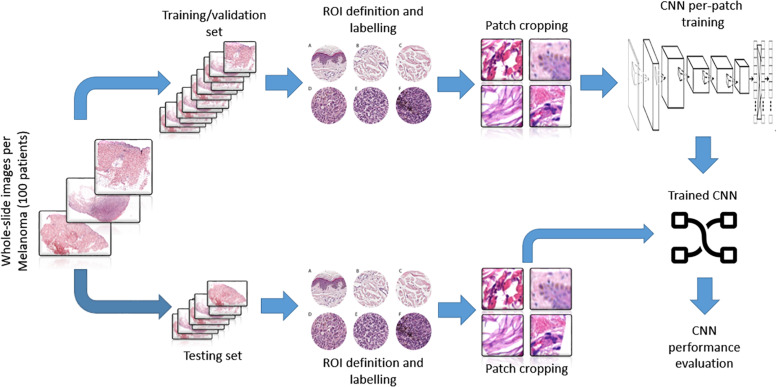
Representative scheme of the methodological pipeline. The whole slide images of melanoma from 100 patients were split in training/validation and testing datasets that are disjointed during the process. ROIs of normal and pathological tissues were extracted from the two datasets and labeled. **(A–C)** Healthy tissue, **(D–F)** invasive cutaneous melanoma. Each ROI was tiled in 299 × 299 pixel patches. The patches of training/validation sets were used for the training of CNN. Finally, the performance indices of classification were estimated by applying the trained CNN to the testing set.

The trained net as a sliding window over the whole WSI was applied to evaluate the topological distribution of the melanoma and healthy tissue. A scheme of the methodological process, from raw WSI to trained CNN performance evaluation, is reported (Figure 3).

## Results

**FIGURE 4:**
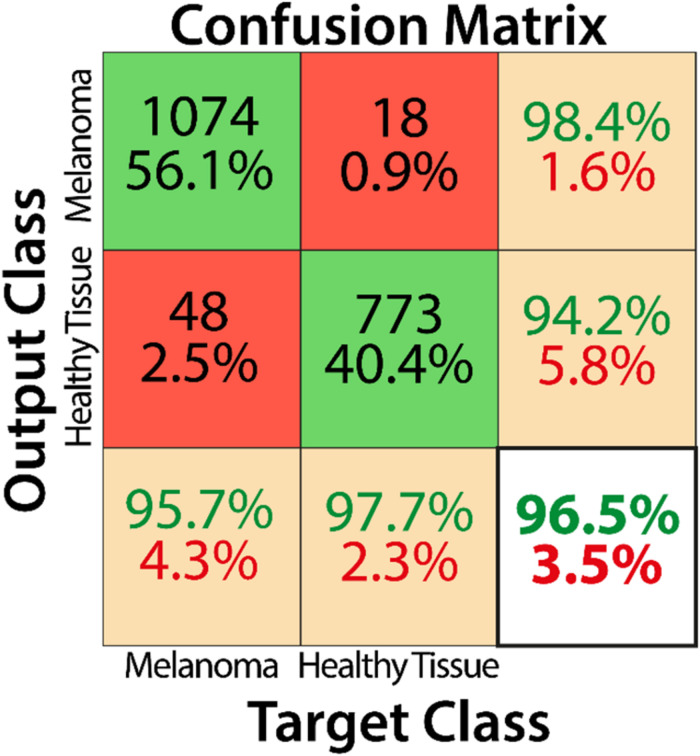
Confusion matrix describing the classification performance of the trained CNN for the training dataset.

Training by using the curated image patches took approximately 18 h to complete 3200 iterations with Matlab software (R2019b, Natick, MA, United States: The MathWorks Inc.) and its Deep Learning Toolbox. As reported in the confusion matrix (Figure 4), the overall accuracy of our net in training set classification was 96.5% (1847 correct classification from a total of 1913). In particular, the misclassification rates were 2.3% for healthy tissue (18 patches of 791) and 4.3% for melanoma (48 patches of 1122). The obtained sensitivity, specificity, and F_1_ score were 95.7, 97.7, and 97.0%, respectively, and Cohen’s kappa was 0.929.

**FIGURE 5:**
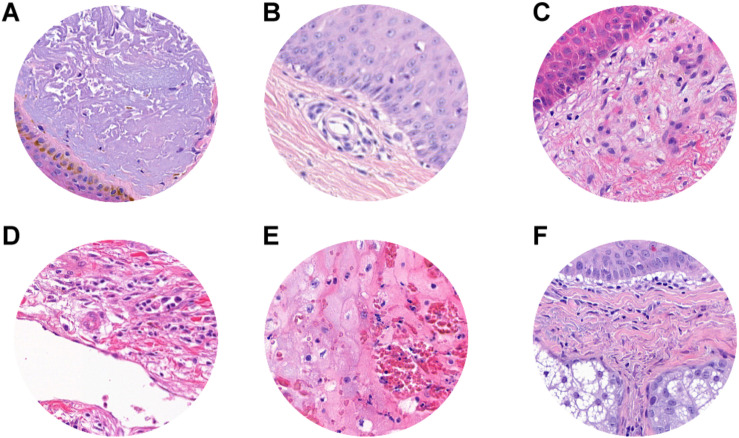
ROI samples in which false-positive patches were identified. **(A)** Dermal solar elastosis (Grade 3). **(A–C)** Epidermal atrophy. **(D)** Abnormal dilated small-to-medium size vessels surrounded by normal dermal collagen. **(D,E)** Extravasated erythrocytes in the dermis or within adnexal epithelium. **(F)** Sebaceous adnexal structures and follicular epithelium.

Misclassified patches were reviewed, and we found that, in 2/9 false-positive analyzed patches, signs of marked dermal solar elastosis (grade 3) (Figure 5A) and/or epidermal atrophy (Figures 5A–C) were observed. In the false-positive cases, the prevalence of dermal-based, instead of epidermal based or dermo-epidermal, patches was observed. In particular, in 3/9 of false-positive patches, an abnormal dilated small-to-medium-size vessel surrounded by normal dermal collagen was observed (Figure 5D). Extravasated erythrocytes in the dermis or within the adnexal epithelium were found in 3/9 cases (Figures 5D,E), and, in one case, normal sebaceous adnexal structures and follicular epithelium were detected (Figure 5F).

**FIGURE 6:**
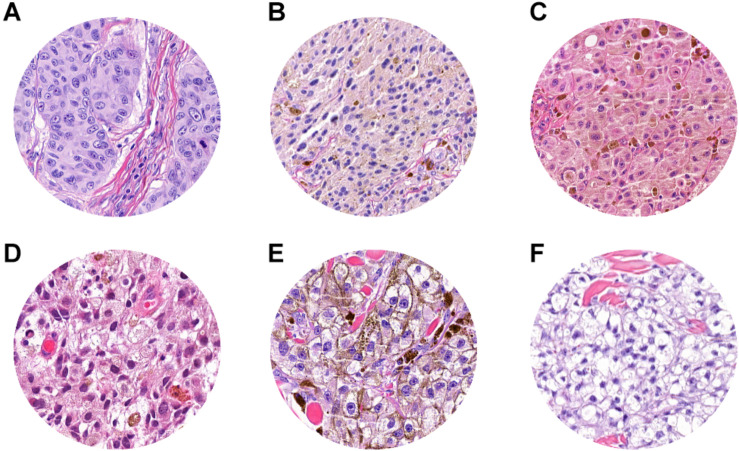
ROI samples in which false-negative patches were identified. **(A)** Absent pigmentation. **(B–D)** Mild pigmentation. **(E)** Prominent pigmentation. **(F)** Clear cell changes in melanoma cell.

Histopathological reevaluation of the false-negative cases showed representative areas of pathological tissue with moderate-to-severe cytological atypia (melanoma cells) in all cases, with no significant areas of non-tumoral tissues. A known factor that negatively affects tumor classification by CNN is the presence of melanin (21); in our dataset, we observed that the level of pigmentation in false negatives was heterogeneous. In particular, it was absent in 5 cases (Figure 6A), mild in 8 cases (Figures 6B–D), and prominent (Figure 6E) in 18 cases, thus demonstrating that the trained neural network was not influenced by the presence or absence of melanin. Clear cell changes were detected in melanoma cells in two patches (Figure 6F). A more detailed analysis showed that, in 7/18 cases, tumor cells were arranged in large confluent aggregates (diffuse growth), with no nests. Tumor-infiltrating lymphocytes (TILs) were absent (16/18) or non-brisk (2/18), and none of the selected images showed a prominent (brisk) infiltration by TILs.

**FIGURE 7:**
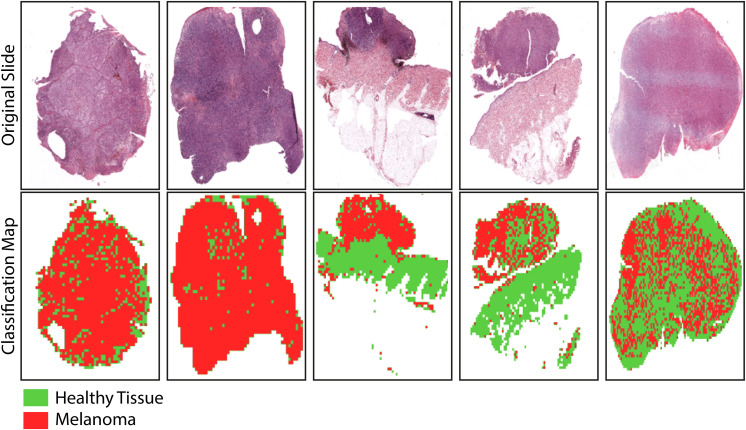
Sample images showing the results of the trained-CNN application to five WSIs. Each WSI was associated with a classification binary map with texels representing classified patches: green area for non-melanoma patches and red area for melanoma patches. The background areas (corresponding to patches with more than 50% of the white background) were represented by the white areas.

Finally, the classification maps obtained after the application of the trained net to five representative WSIs of invasive cutaneous melanoma were reported (Figure 7). The maps showed the topological distribution of patches of each WSI classified as healthy tissue (green area) or melanoma (red area).

## Discussion

Since its development in the mid-twentieth century, research using AI has been subjected to transformation and criticism. Using a powerful workstation, a large amount of data, and complex computer algorithms, AI can identify complex models in the real world, producing considerations, conclusions, extrapolations, associations, and forecasts that can match or exceed human capabilities. The analysis of histopathological images in general, and melanoma in particular, represent a natural application of this field.

In this study, we showed that an AI algorithm to recognize cutaneous melanoma can be a useful diagnostic tool for supporting dermatopathologists for diagnostic purposes. Although extensive research has been carried out on melanoma dermatoscopic images processing with AI (22–24), few studies have specifically shown the utility of AI in the recognition of melanoma compared to normal tissue in histopathological images (17–19).

Previous studies (18, 19) have proposed CNNs to distinguish between histopathological images of melanomas and benign tissue. First, a pretrained ResNet50 CNN with a randomly cropped area from 595 WSIs (300 nevi WSIs for 50152 patches; 295 melanoma WSIs for 55263 patches) and tested with a randomly cropped area from 100 WSIs (50 nevi WSIs for 50152 patches; 50 melanoma WSIs for 55263 patches) was used, and an overall accuracy of 81% with respect to the gold standard reference (i.e., classification by board-certified pathologists) was reported (18). Second, a CNN that automatically detected melanoma in WSIs and highlighted the lesion area on WSIs using a probabilistic heat map was reported by using a pretrained VGG16 CNN, trained with 38 WSI (27 healthy tissue WSIs for 50152 patches; 11 melanoma WSI for 55263 patches), and tested with an independent dataset of 76 WSIs (38 healthy tissue WSIs for 16904 patches; 38 melanoma WSI for 66222 patches). For patch diagnosis on an independent dataset, the proposed model also achieved accuracy, sensitivity, and specificity of 91.4, 91.0, and 92.8%, respectively (19).

Our data clearly show that the AI algorithm we built includes all the characteristics necessary for better recognition of tumor cells, including lesions that are not easily identified. The CNN proposed in this study has shown high performance in detecting cutaneous melanoma areas in histopathological slides. It can recognize portions of pathological and healthy tissues on independent testing datasets with an accuracy, sensitivity, specificity, and F_1_ score of 96.5, 95.7, 97.7, and 97.0%, respectively. Unlike recently published data (18), our classification method does not aim to discriminate between melanoma and nevi, but rather to distinguish, within a WSI, healthy tissue from pathological tissue. Our aim is in line with the patch-level classification on an independent dataset (19). In addition, we obtained a classification performance higher than that reported (19), in terms of accuracy (96.5% vs. 91.4%), sensitivity (95.7% vs. 91.0%), and specificity (97.7% vs. 92.8%). In the previous study (19), the F_1_ score was not estimated and, therefore, we cannot compare it with the F1 score of our net; nevertheless, the value obtained is high enough to demonstrate the high classification accuracy of our CNN.

The proposed CNN has the potential to quickly classify and give more detailed information on pathological cases, defining a heat map that distinguishes the malignant areas from the normal ones. A promising application would be for the pathologist to focus on a more accurate and faster identification of the tumor margin status, thus facilitating a heat map on the scan, to streamline the task in clinically critical decisions. Our results have also revealed that a dimension of about 5000 mm^2^ per patch is sufficient to obtain a reliable patch-level recognition of pathological tissue in WSIs. The proposed CNN achieved a Cohen’s kappa of 0.929 with respect to the reference classification of expert dermatopathologists, thus showing a high level of agreement between human and machine evaluation, as well as a higher inter-rater agreement with respect to the previous study (19), which reported a Cohen’s kappa of 0.878 for patch-level classification on an independent dataset.

In our study, each misclassified patch was visually reanalyzed by expert dermatopathologists in search of possible explanations for the wrong classification by the trained CNN. In some of the false-positive patches, signs of marked dermal solar elastosis and epidermal atrophy, as expected from UV-related melanomas arising on chronically sun-exposed anatomical sites, were observed. This finding is significant in light of the recent multidimensional classification for melanoma (25). The possibility that the AI algorithms implemented allow a better subclassification of Low-Cumulative Solar Damage (CSD) melanomas vs. High-CSD melanomas, which are different, as well as the genomic level and mutational status, should be further explored in larger data sets. In addition, a misdiagnosis could be attributed to the presence of more heterogeneous patches showing prominent dilated vessels and extravasated erythrocytes, or adnexal structures in the dermis. In all false-negative patches, areas of homogeneous pathological tissue with moderate–severe cytological atypia (melanoma cells), with no significant areas of non-tumoral tissues, were recognized. In about half of the cases, tumor cells were arranged in large confluent aggregates with no discrete nests. Whether or not the type of architectural tumor growth and cellular arrangement (confluent aggregates vs. small discrete nests) affects misclassification is currently a matter of investigation. None of the false-negative patches were associated with brisk TILs, thus avoiding the hypothesis that a prominent tumor-associated lymphocytic component in the tumor microenvironment could be responsible for the misclassification. Finally, tissue pigmentation did not affect classification.

The strength of our study is in the description of an AI that is proficient in working on a heterogeneous data set of melanomas, which reflects the cases usually inspected by dermatopathologists in daily clinical practice. However, this study has several limitations that will be addressed in future studies. First, the case series includes only pT3 and pT4 melanomas (Breslow thickness > 2 mm). Indeed, the purpose of this study was to develop a CNN that works with high performance on thick melanomas; the second step will be to apply the neural network to melanomas with Breslow <2 mm, to evaluate its performance and, possibly, implement training with thinner melanomas. Second, a separate cohort of melanocytic nevi was not included in the comparison; we acknowledge that the ability to discriminate melanoma from nevi would increase the strength of the proposed AI approach. Third, additional studies incorporating larger datasets from clinical practice settings, as well as more general pathologists with a broader range of backgrounds, are necessary to further validate our data. Finally, the technical requirements for image acquisition should be validated in additional independent cohorts. In particular, the classification performance of the net could be reduced by the use of different digital WSI scanners.

## Conclusion

In conclusion, Our data show that a deep learning system can be trained to recognize melanoma samples, achieving accuracies comparable to experienced dermatopathologists. This system could prove to be a valuable aid in improving diagnostic efficiency when expert consultation is not available, as well as reducing interobserver variability. Further studies in larger data sets are required to verify whether the deep learning algorithm allows subclassification of different melanoma subtypes.

## Members of the Italian Association for Cancer Research (AIRC) Study Group

The Italian Association for Cancer Research (AIRC) Study Group includes the following members who participated in this study and should be considered as co-authors: Michele Maio (MM), Azienda Ospedaliera Universitaria Senese, Siena, Italy. Andrea Anichini (AA), Istituto Nazionale Tumori, Milan, Italy. Giuseppe Palmieri (GP), Institute of Genetic and Biomedical Research, National Research Council, Sassari, Italy. Ulrich Pfeffer (UP), IRCCS Ospedale Policlinico San Martino, Genoa, Italy.

## Data Availability Statement

The raw data supporting the conclusions of this article will be made available by the authors, without undue reservation.

## Ethics Statement

The studies involving human participants were reviewed and approved by the use of FFPE sections of human samples and the Local Ethics Committee (#13676_bio and #17033_bio) according to the Declaration of Helsinki. The patients/participants provided their written informed consent to participate in this study.

## Author Contributions

FD, FU, ML, RN, and DM: conceptualization, writing the original draft, and funding acquisition. DM: funding acquisition. ML: methodology. VM and SS: tissue collection and methodology. FD, FU, ML, RN, AC, and DM: review of draft. FD, FU, and ML: editing the draft. The Italian Association for Cancer Research (AIRC) Study Group, Michele Maio (MM), Andrea Anichini (AA), Giuseppe Palmieri (GP), Ulrich Pfeffer (UP), DM, and AC: resources. All authors contributed to the article and approved the submitted version.

## Conflict of Interest

The authors declare that the research was conducted in the absence of any commercial or financial relationships that could be construed as a potential conflict of interest.

## Abbreviations

AI: artificial intelligence
CNNs: convolutional neural networks
FFPE: formalin fixed paraffin embedded
TIL: tumor infiltrating lymphocytes
WSIs: whole-slide images.
